# CHINESE MEDICINE (CM) matters

**DOI:** 10.1186/1749-8546-3-16

**Published:** 2008-11-28

**Authors:** Chun Un, Lorita Cheang, Jerry Lei, Siu-wai Leung, Hin Wing Yeung

**Affiliations:** 1 International Society for Chinese Medicine (ISCM), A & C, 1st Floor, Block 2, University of Macau, Av. Padre Tomas Pereira, Taipa, Macao, PR China

## 

Happy birthday CM! On 23 November 2006, we launched *Chinese Medicine *(CM), a peer-reviewed, open access, international, interdisciplinary and scholarly journal in Chinese medicine [1]. Two years on, with the best efforts and sometimes personal sacrifices made by the authors, editorial and advisory board members, reviewers, and the editorial teams in Macao and at BioMed Central in London, we have published a total of thirty-two high quality research and review articles. In addition, four print issues have also been distributed to many individuals and institutions worldwide.

Two years ago, we committed ourselves to publishing valuable research in Chinese medicine. We appreciate all rigorous research methodologies and evidence, whether they are traditional or modern, conventional or alternative, macroscopic or microscopic. All articles (except editorials) published in CM must be peer-reviewed to ensure that they are evidence-based, scientifically justified and ethical. A total of thirteen review articles and eighteen research articles and one editorial have been published. They are important contributions to Chinese medicine, covering a wide range of topics/interests such as acupuncture, *materia medica*, Chinese medicine formulae, dentistry, allergy, metabonomics, genomics, cardiovascular diseases, liver diseases, cancer, arthritis, ageing, quality control and drug safety, and more. They have been highly accessed and widely cited. As they are indexed and permanently archived by major international scientific repositories and web-based search engines for e-journals such as PubMed, PubMed Central, Potsdam, INIST, e-Depot, CAS, CABI, Citebase, OAIster, Cinahl, Scopus, Scirus, Google Scholar and Zetoc, the general public as well as Chinese medicine researchers can always access them for information and reference.

Not surprisingly, CM published articles on ginseng – a hot research topic. Yue *et al*. [2] relate the pharmacogenomics of ginseng to 'Yin and Yang' actions, particularly the anti-tumor, angiomodulating and steroid-like activities of ginsenosides. Mechanistic and functional genomic studies revealed that a group of genes were regulated by ginsenosides in endothelial cells and ginsenosides (Rb1 and Rg1) with opposing activities. Moreover, the authors demonstrate that ginsenosides can act as functional ligands to activate different steroid hormone receptors. Such research findings may help discover the mystery of the diverse pharmacological activities and therapeutic effects of ginseng. The 'Ying and Yang' actions of ginseng, particularly ginsenosides (Re and Rg3), as reported by Ng *et al*. [3], alter cellular redox state of a tumour cell model in opposite directions. Equally fascinating accounts are given by Ko *et al*. [4] relating the ancient ideas of Yin and Yang in Chinese medicine to the biological concepts of ATP generation, antioxidant and immunomodulation. Wu *et al*. [5] also introduce us to the modulating effects of American ginseng on pancreatic beta cell activities.

As a natural and holistic approach, traditional Chinese herbal medicine often uses formulae consisting of multiple herbs. The complexity of Chinese medicine formulae is a big technical challenge to the research and quality assurance/control of herbal products. CM has published six articles to address this issue including the compound-oriented and pattern-oriented approaches [6], DNA methods for identification of Chinese medicinal materials [7], chemical markers for the quality control of herbal medicines [8], the applications of high-performance liquid chromatograph (HPLC) method in the quality assurance of complex Chinese medicine formulae *Dachenggi Tang *[9], *Gegen Tang *granule [10] and herbal products of *Radix Aconiti Lateralis Perarata *[11]. CM has also published several studies on the complexity of multiple herb formulae, e.g. a systematic approach to revealing the complexity of Chinese herbal medicine formulae [12], an animal study of *Yinchenhao Tang *on cirrhosis/fibrosis [13] and a clinical study on *Duhuo Jisheng Tang *for treating osteoarthritis [14].

Clinical studies in Chinese medicine are often not well reported. We have been trying to improve the situation by enforcing the international standards of clinical trial reporting in our published articles. Capodice *et al*. [15] and Itoh *et al*. [16] report the applications of acupuncture to treat lower urinary tract symptoms related to chronic prostatitis/chronic pelvic pain and knee osteoarthritis respectively. Another clinical study on treating knee osteoarthritis with Chinese herbal medicine is reported by Lai *et al*. [14]. A meta-analysis by Liu *et al*. [17] assesses the effectiveness and safety of Chinese red yeast rice preparations on lipid modification in primary hyperlipidemia. A systematic review by Liu *et al*. [18] investigates the beneficial effects of green tea on caner prevention by evaluating forty-three epidemiological studies, four randomized trials and one meta-analysis.

In a review article, Wang *et al*. [19] survey the literature for a list of Chinese herbal ingredients with reported liver protection activities, in which a total of 274 different species and hundreds of active ingredients were examined. In another review article, Adams *et al*. [20] discuss the pharmacology, medicinal chemistry and clinical studies published for *danshen *and tanshinone preparations. Itokawa *et al*. [21] survey the literature between 1976 and mid-2008 on the anti-inflammatory, anti-oxidant, anti-HIV, chemopreventive and anti-prostate cancer effects of curcuminoids.

On the front of the so-called 'omics', Qiu *et al*. [22] demonstrate that metabolic profiling is useful in studying therapeutic mechanisms of herbal medicines such as *Herba Cistanches*. The Faculty of Dentistry of the University of Hong Kong has contributed two articles to CM on bone formation authored by Wong *et al*. [23,24]. We hope that this series of papers will bear the fruit of a newly proposed research discipline, dubbed as Evidence-based Integrative Dentistry (EBID). CM encourages submissions in this emerging field.

Two years ago, we committed ourselves to the internationalization and modernization of Chinese medicine [1]. Today, we boast a much internationalized authorship in the Chinese medicine field. The authorship of the published articles covers thirteen countries and regions. We are also proud of the support from the leading scholars and experts in Chinese medicine and integrative medicine serving as our advisory/editorial board members and/or our reviewers.

As of 28 November 2008, the acceptance rate of CM during this two-year period has been 20.13%. In our editorial work, we recognize a great demand for improving English writing skills that are crucial to reporting research findings. We have been providing our authors with extensive English writing assistance and copy-editing, which is why some papers took longer time to publish. We are also planning a scientific writing workshop to help the authors in need.

Two years ago, we committed ourselves to Open Access to 'ensure that the best Chinese medicine research is published and read by the widest possible range of international audience' and that 'in a speedy and professional manner, the scientific value and potential impact of all published articles in CM will be judged and evaluated by the international scientific community' [1]. Today, access and citation data speak for themselves. As of 28 November 2008, the two most viewed articles of all time have recorded access numbers of 14,527 and 10,396 respectively. Eleven out of the thirty-two articles published have been cited and the total number of citations has amounted to 36. The two most cited articles have been cited eight and five times respectively. Recently published articles have also been cited. The access and citation data show that the articles published in CM have high international visibility and accessibility and are tremendously citation friendly as stated in our launch editorial [1]. The data also support that open access articles are immediately read and cited by peers, leading to a quicker and higher number of citations [25-27].

For the future, we strongly encourage our authors to follow the international guidelines for scientific reporting when they submit a manuscript, while rigorous reviews of manuscripts must also be upheld. We firmly believe that the synergy generated by the authors, reviewers and editors will enable this journal to publish more and better papers. We are more convinced now than two years ago that this journal is an exciting medium for fostering open access strengths and evidence-led forces for the advancement of Chinese medicine [1].

## Competing interests

The authors declare that they have no competing interests.

## Authors' contributions

CU drafted the first version of the manuscript for discussion. Other authors contributed their views and revised the manuscript. HWY led the revision process, integrated all views and finalized the manuscript. All authors read and approved the final manuscript.
